# Second primary cancer after primary peritoneal, epithelial ovarian, and fallopian tubal cancer: a retrospective study

**DOI:** 10.1186/s12885-018-4700-3

**Published:** 2018-08-08

**Authors:** Myong Cheol Lim, Young-Joo Won, Jiwon Lim, Tahereh Salehi, Chong Woo Yoo, Robert E. Bristow

**Affiliations:** 1 Division of Gynecologic Oncology, Obstetrics and Gynecology, Irvine Medical Center, University of California, California, USA; 2 Center for Uterine Cancer and Center for Clinical Trials, Hospital, National Cancer Center, Goyang, Republic of Korea; 3 Cancer Healthcare Research Branch, Research Institute, National Cancer Center, Goyang, Republic of Korea; 4 Department of Cancer Control & Population Health, Graduate School of Cancer Science and Policy, National Cancer Center, Goyang, Republic of Korea; 5 Cancer Registration and Statistics Branch, National Cancer Control Institute, National Cancer Center, Goyang, Republic of Korea

**Keywords:** Second primary, Ovarian cancer, Primary peritoneal cancer, Fallopian tubal cancer

## Abstract

**Background:**

In this retrospective study, data from patients listed in the Korea Central Cancer Registry during 1993–2014 were analysed, to investigate the incidence and survival of second primary cancers (SPCs) after a diagnosis of primary peritoneal, epithelial ovarian, and fallopian tubal (POFT) cancer.

**Methods:**

The standardised incidence ratio (SIR) and survival outcomes of patients with SPCs among POFT cancer survivors were analysed.

**Results:**

Among 20,738 POFT cancer survivors, 798 (3.84%) developed SPCs, at an average interval of 5.50 years. SPC risk in POFT survivors (SIR, 1.29) was higher compared to the general population. The most high-risk type of SPC was leukaemia (3.07) followed by the lung and bronchus (1.80), colon (1.58), rectum and rectosigmoid junction (1.42), thyroid (1.34), and breast (1.26). In women aged < 60 years, cancer of the breast (1.30), ascending colon (2.26), and transverse colon (4.07) as SPCs increased. Up to 10 years after POFT cancer treatment, leukaemia risk increased, especially in those < 60 years, with serous histology, and with distant stage, which required aggressive chemotherapy. The median overall survival time was 12.8 years and 14.3 years in women with POFT cancer and SPCs, respectively. Thyroid and breast cancers were favourable prognostic markers among SPCs.

**Conclusions:**

The overall SPC risk increases in POFT cancer survivors, especially in those < 60 years. The cancer risk of breast and the proximal colon increase based on hereditary predisposition, while leukaemia likely develops from aggressive treatment. The median overall survival is favourable in POFT cancer survivors with SPCs.

**Electronic supplementary material:**

The online version of this article (10.1186/s12885-018-4700-3) contains supplementary material, which is available to authorized users.

## Background

Ovarian cancer is one of the most important gynaecologic cancers. In 2017, there were an estimated 22,440 new cases and 14,080 ovarian cancer deaths in the US [1]. The incidence of ovarian cancer has been continuously increasing, resulting in an annual percent change of + 1.5% during 1999–2010, with an estimated incidence of 2618 and mortality of 1168 in 2017 in Korea [2, 3]. The high rate of mortality has not improved in the last decade, and survivors of ovarian cancer may further develop a second primary cancer (SPC) based on the shared aetiology and treatment sequelae.

Ovarian cancer, diagnosed as C56 by the International Statistical Classification of Diseases and Related Health Problems (ICD), includes epithelial cancer and germ cell cancer among others, and is not a single disease entity in terms of the aetiology, treatment strategies, and prognosis. Basically, epithelial ovarian cancer is the same disease entity as primary peritoneal cancer (C48.2) and fallopian tubal cancer (C57). Accordingly, primary peritoneal, epithelial ovarian, and fallopian tubal (POFT) cancers are diagnosed and treated in similar ways.

POFT cancers have shared genetic backgrounds as parts of hereditary cancers related to *BRCA1* and *BRCA2* mutations and Lynch syndrome [4]. Moreover, adjuvant chemotherapy after cytoreductive surgery may be associated with chemotherapy-related SPCs. Therefore, the objective of this study was to investigate the patterns and treatment outcomes according to the development and type of SPC after POFT cancers.

## Methods

Analysis of data from the Korea Central Cancer Registry from 1993 to 2014 identified 20,738 patients with POFT cancers. The methodology for this study on SPC after POFT, in terms of the statistical analysis and expression of the results, is the same as in our previous study on SPC after cervical cancer [5]. The methods are described below to help the readers understand this study.

In brief, the standardised incidence ratios (SIRs) and corresponding 95% confidence intervals (CIs) of SPC among POFT survivors were analysed to quantify the relative risk compared to women in the general population. These SIRs were calculated by dividing the observed number of SPCs by the expected SPC number if the patients in the cohort demonstrated cancer rates equivalent to those for individuals in the general population.

The number of person-years at risk (PYRs) was defined from 2 months after the date of the POFT diagnosis to the date of death or the end date of this study, whichever occurred first. For each initial cancer site grouping, the PYRs and observed cases of cancer were stratified according to 5-year age groups and calendar year. The cancer incidence rates were computed for each subsite of cancer and according to age and calendar year and were multiplied by the accumulated PYRs to estimate the expected number of subsequent cancers for each stratum.

Kaplan-Meier survival curves were calculated for POFT patients with or without an SPC. The differences between the groups were assessed using the log-rank test. All statistical tests were two-sided, and the significance was set at an alpha level of 0.05. To compute the SIRs and their 95% CIs, we used the “MP-SIR” setting of SEER*Stat 8.3.4. Survival curves were generated and log rank-tests were performed using Stata 11 software (StataCorp. 2009, College Station, TX).

## Results

A total of 20,738 survivors who were diagnosed with POFT cancer were evaluated for a mean follow-up period of 5.68 ± 5.33 years (Table 1). The mean age at the initial diagnosis of POFT was 51.18 years. The incidence of POFT diagnosis peaked during the ages 50–59 years (28.38%). Of the 20,738 POFT survivors, 798 (3.84%) survivors developed an SPC. The mean interval from the initial POFT diagnosis to the SPC was 5.5 years (±4.71), and the mean age at diagnosis with the SPC was 56.00 years (±11.88).

**Table 1:** Characteristics of patients with primary POFT cancer

Variable	Number	Percent
Women with POFT cancer	20,738	100.00
Peritoneal cancer	506	2.44
Epithelial ovarian cancer	19,767	95.32
Fallopian tubal cancer	465	2.24
Average follow-up, years (mean, SD)	5.68	5.33
Average age at diagnosis of POFT cancer, years (mean, SD)	51.18	13.37
Age at diagnosis of the 1st primary cancer, years		
< 30	1346	6.49
30–39	2394	11.54
40–49	5520	26.62
50–59	5885	28.38
60–69	3763	18.15
70–79	1597	7.70
≥ 80	233	1.12
Histology		
Serous	11,793	56.87
Mucinous	4332	20.89
Endometrioid	2304	11.11
Clear cell	1929	9.30
Others	380	1.84
Stage^*^		
Localized	3378	28.70
Regional	2365	20.09
Distant	5416	46.01
Unknown	612	5.20
Women who developed SPC	798	3.84
Average interval between first POFT cancer and SPC, years (mean, SD)	5.50	4.71
Average age at diagnosis of SPC, years (mean, SD)	56.00	11.88

^*^Stage data was used since 2006

POFT: peritoneal, epithelial ovarian, and fallopian tubal; SPC: second primary cancer; SD, standard deviation

As shown in Table 2, the overall SIR for an SPC was 1.29 (95% CI, 1.21–1.38). The most high-risk type of SPC was leukaemia (3.07), followed by cancer of the lungs and bronchus (1.8), colon (1.58), rectum and rectosigmoid junction (1.42), thyroid (1.34), and breasts (1.26). The SIR of an SPC was higher in young (age < 60 years) women (1.47; 95% CI, 1.36–1.59). In these young survivors, the types of highest SPC risk were leukaemia (3.86), cancer of the lungs and bronchus (2.61), colon (2.00), rectum and sigmoid junction (1.67), thyroid (1.35), and breasts (1.30). Of the cases of colon cancer in young women, high observed-to-expected ratios were observed in transverse colon (4.07) and ascending colon cancers (2.26).

**Table 2:** Risk of second primary cancer after primary POFT cancer diagnosis by age and follow-up period

	Total			Age (years)		Follow-up (months)		
				< 60	≥60	2–59	60–119	≥120
	SIR	O/E	CI	SIR	SIR	SIR	SIR	SIR
Leukaemia	3.07^#^	(22/7.16)	(1.92–4.65)	3.86^#^	1.60	3.16^#^	3.68^#^	2.05
Urinary bladder	1.99	(10/5.03)	(0.95–3.66)	2.90^#^	1.35	2.01	2.93	0.85
Lung, bronchus	1.80^#^	(81/44.89)	(1.43–2.24)	2.61^#^	1.01	1.26	2.64^#^	1.99^#^
Rectum, rectosigmoid junction	1.42^#^	(47/33.04)	(1.05–1.89)	1.67^#^	1.08	1.53	1.35	1.27
Thyroid	1.34^#^	(206/153.16)	(1.17–1.54)	1.35^#^	1.32	1.57^#^	1.21	0.95
Colon	1.58^#^	(122/77.38)	(1.31–1.88)	2.00^#^	1.03	1.69^#^	1.34	1.60^#^
Ascending colon	1.77^#^	(25/14.14)	(1.14–2.61)	2.26^#^	1.28	1.60	1.31	2.61^#^
Transverse colon	2.43^#^	(14/5.77)	(1.33–4.07)	4.07^#^	0.71	1.79	1.93	4.22^#^
Descending colon	1.17	(23/19.64)	(0.74–1.76)	1.43	0.77	1.52	1.50	0.00^#^
Rectum	1.45^#^	(48/33.13)	(1.07–1.92)	1.72^#^	1.08	1.52	1.34	1.40
Other and unspecified	2.55^#^	(12/4.71)	(1.32–4.45)	4.01^#^	1.21	3.74^#^	0.00	2.94
Female breast	1.26^#^	(128/101.85)	(1.05–1.49)	1.30^#^	0.99	1.29^#^	1.47^#^	0.87
Kidney parenchyma	1.43	(12/8.37)	(0.74–2.50)	1.49	1.34	1.64	1.78	0.54
Renal pelvis, other urinary	2.38	(5/2.10)	(0.77–5.56)	2.31	2.43	3.02	0.00	3.69
Pancreas	1.25	(20/15.94)	(0.77–1.94)	1.72	0.89	0.91	2.55^#^	0.51
Non-Hodgkin lymphoma	0.88	(11/12.43)	(0.44–1.58)	1.13	0.45	1.09	0.60	0.74
Bile ducts, other biliary	1.43	(26/18.23)	(0.93–2.09)	2.20^#^	0.86	1.57	1.01	1.61
Stomach	0.89	(67/75.35)	(0.69–1.13)	1.15	0.55^#^	0.56^#^	1.34	1.15
Liver	0.68	(16/23.54)	(0.39–1.1)	0.81	0.50	0.71	0.48	0.86
Gallbladder	0.64	(6/9.43)	(0.23–1.38)	1.21	0.19	0.64	0.39	0.92
Small intestine	1.93	(4/2.07)	(0.53–4.94)	3.37	0.00	1.89	0.00	4.38
All excluding POFT	1.29^#^	(838/647.47)	(1.21–1.38)	1.47^#^	0.91	1.33^#^	1.38^#^	1.11

POFT: peritoneal, epithelial ovarian, and fallopian tubal; SIR: standardised incidence ratio; O/E: observed/expected; CI: confidence interval

^#^significant at α = 0.05

Up to 10 years after the initial diagnosis of POFT cancer, the risk of SPCs increased (1.33 until 5 years and after then, 1.38 until 10 years). During the first 5 years of follow-up, the SPC type with the highest SIR was leukaemia (3.16), followed by cancer of the colon (1.69), thyroid (1.57), and breast (1.29). The risk of stomach cancer significantly decreased (0.56) during the first 5 years of follow-up. During 6–10 years of follow-up after the diagnosis of POFT cancer, the overall SIR was 1.38 (95% CI, 1.21–1.57). The cancer types with the highest SIR were leukaemia (3.68; 95% CI, 1.48–7.59), lung and bronchus (2.64), pancreas (2.55), and breast (1.47). After 10 years of follow-up, the observed-to-expected ratio of colon cancer increased again (1.60), owing to the increased risks of transverse colon cancer (4.22) and ascending colon cancer (2.61) (Table 2).

In the serous histologic subgroup (Table 3), the risks of leukaemia (4.77) and breast cancer (1.58) were high, whereas the risk of neither cancer was increased in the other histology subgroups, including mucinous, endometrioid, clear, and transitional cell POFT cancers. When the POFT cancers were divided by stage (Table 4), the risk of leukaemia increased in those with distant stage (4.94), but not localized and regional stages. The risk of leukaemia as an SPC after POFT cancer was higher among patients receiving chemotherapy; however, this relationship did not reach statistical significance (hazard ratio, 2.00; *p* = 0.17).

**Table 3:** Risk of second primary cancer after primary POFT cancer diagnosis according to histology

Histology	Serous		Mucinous		Endometrioid		Clear cell		Transitional cell		Carcinosarcoma	
	SIR	CI	SIR	CI	SIR	CI	SIR	CI	SIR	CI	SIR	CI
Leukaemia	4.77^#^	(2.83–7.54)	1.14	(0.14–4.11)	0.00	(0.00–3.95)	3.48	(0.42–12.58)	0.00	(0.00–37.27)	0.00	(0.00–127.79)
Urinary bladder	1.81	(0.59–4.22)	1.68	(0.20–6.06)	4.59	(0.95–13.43)	0.00	(0.00–11.77)	0.00	(0.00–51.86)	0.00	(0.00–128.39)
Lung, bronchus	1.08	(0.71–1.58)	3.59^#^	(2.52–4.98)	1.54	(0.70–2.92)	2.51^#^	(1.08–4.94)	1.52	(0.0–8.47)	0.00	(0.00–15.84)
Rectum, rectosigmoid junction	1.54^#^	(1.03–2.23)	1.22	(0.56–2.32)	1.84	(0.79–3.62)	0.39	(0.01–2.19)	2.06	(0.05–11.5)	0.00	(0.00–24.49)
Thyroid	1.27^#^	(1.03–1.55)	1.26	(0.93–1.68)	1.77^#^	(1.24–2.45)	1.17	(0.70–1.83)	2.86^#^	(1.05–6.22)	0.00	(0.00–9.17)
Colon	1.31	(0.99–1.7)	1.82^#^	(1.23–2.58)	2.56^#^	(1.67–3.75)	0.85	(0.28–1.98)	2.62	(0.54–7.65)	2.75	(0.07–15.33)
Ascending colon	1.01	(0.44–1.99)	2.93^#^	(1.34–5.57)	2.73	(0.89–6.37)	1.97	(0.24–7.11)	0.00	(0.00–17.3)	14.02	(0.35–78.11)
Transverse colon	0.62	(0.08–2.24)	3.97^#^	(1.29–9.27)	6.68^#^	(2.17–15.59)	4.77	(0.58–17.24)	0.00	(0.00–42.6)	0.00	(0.00–126.37)
Descending colon	1.01	(0.51–1.81)	1.41	(0.52–3.07)	1.94	(0.63–4.52)	0.00	(0.00–2.32)	3.40	(0.09–18.96)	0.00	(0.00–42.32)
Rectum	1.59^#^	(1.07–2.29)	1.22	(0.56–2.32)	1.83	(0.79–3.61)	0.39	(0.01–2.18)	2.06	(0.05–11.47)	0.00	(0.00–24.43)
Other and unspecified	2.34	(0.86–5.09)	1.78	(0.22–6.45)	4.84	(1.00–14.14)	0.00	(0.00–11.74)	15.06	(0.38–83.91)	0.00	(0.00–148.5)
Female breast	1.58^#^	(1.26–1.97)	0.78	(0.47–1.22)	1.38	(0.83–2.16)	0.48	(0.16–1.12)	1.42	(0.17–5.14)	3.60	(0.09–20.08)
Kidney parenchyma	1.75	(0.75–3.44)	1.09	(0.13–3.95)	0.00	(0.00–3.35)	2.85	(0.34–10.29)	0.00	(0.00–29.83)	0.00	(0.00–104.36)
Renal pelvis, other urinary	3.37	(0.92–8.63)	2.15	(0.05–11.98)	0.00	(0.00–13.70)	0.00	(0.00–27.27)	0.00	(0.00–117.11)	0.00	(0.00–310.28)
Pancreas	1.01	(0.46–1.92)	1.95	(0.78–4.02)	1.95	(0.53–4.98)	0.00	(0.00–3.49)	0.00	(0.00–15.72)	0.00	(0.00–42.09)
Non-Hodgkin lymphoma	1.04	(0.42–2.15)	1.06	(0.22–3.09)	0.00	(0.00–2.27)	0.00	(0.00–3.61)	0.00	(0.00–20.58)	19.38	(0.49–108.01)
Bile ducts, other biliary	1.37	(0.75–2.31)	0.72	(0.15–2.11)	1.69	(0.46–4.34)	3.45	(0.94–8.83)	0.00	(0.00–13.89)	9.79	(0.25–54.55)
Stomach	0.88	(0.62–1.22)	1.14	(0.70–1.77)	0.91	(0.42–1.73)	0.36	(0.04–1.28)	0.00	(0.00–3.44)	0.00	(0.00–10.67)
Liver	0.84	(0.42–1.51)	0.19	(0.00–1.08)	0.00	(0.00–1.19)	1.71	(0.35–5.00)	0.00	(0.00–10.68)	9.41	(0.24–52.43)
Gallbladder	0.38	(0.05–1.38)	0.92	(0.11–3.33)	1.63	(0.20–5.87)	0.00	(0.00–6.08)	0.00	(0.00–27.04)	0.00	(0.00–71.35)
Small intestine	2.63	(0.54–7.69)	2.17	(0.05–12.08)	0.00	(0.00–13.56)	0.00	(0.00–23.26)	0.00	(0.00–123.21)	0.00	(0.00–382.12)
All excluding POFT	1.26^#^	(1.15–1.39)	1.26^#^	(1.09–1.45)	1.64^#^	(1.38–1.93)	0.98	(0.74–1.27)	1.31	(0.68–2.29)	2.80^#^	(1.13–5.77)

POFT: peritoneal, epithelial ovarian, and fallopian tubal; SIR: standardised incidence ratio; CI: confidence interval

^#^significant at α = 0.05

**Table 4:** Risk of second primary cancer after primary POFT cancer diagnosis according to stage*

Stage	Localized		Regional		Distant	
	SIR	CI	SIR	CI	SIR	CI
Leukaemia	2.73	(0.33–9.87)	1.96	(0.05–10.92)	4.94^#^	(1.60–11.53)
Urinary bladder	0.00	(0.00–8.53)	0.00	(0.00–11.82)	4.51	(0.93–13.18)
Lung, bronchus	3.46^#^	(1.94–5.71)	0.61	(0.07–2.22)	0.88	(0.32–1.92)
Rectum, rectosigmoid junction	1.89	(0.69–4.11)	0.85	(0.10–3.06)	2.28^#^	(1.14–4.08)
Thyroid	2.38^#^	(1.79–3.10)	1.32	(0.80–2.03)	1.19	(0.82–1.67)
Colon	2.10^#^	(1.20–3.41)	1.58	(0.72–2.99)	1.36	(0.78–2.21)
Ascending colon	2.87	(0.78–7.34)	1.88	(0.23–6.79)	0.45	(0.01–2.48)
Transverse colon	1.72	(0.04–9.56)	0.00	(0.00–8.36)	0.00	(0.00–3.98)
Descending colon	1.94	(0.53–4.97)	1.94	(0.40–5.67)	0.63	(0.08–2.29)
Rectum	1.88	(0.69–4.10)	0.84	(0.10–3.05)	2.27^#^	(1.13–4.06)
Other and unspecified	2.52	(0.06–14.05)	7.03	(0.85–25.38)	3.34	(0.40–12.05)
Female breast	0.99	(0.53–1.69)	1.56	(0.85–2.62)	1.27	(0.79–1.94)
Kidney parenchyma	2.19	(0.27–7.92)	0.00	(0.00–5.45)	2.21	(0.46–6.45)
Renal pelvis, other urinary	0.00	(0.00–18.45)	0.00	(0.00–24.4)	9.17^#^	(1.89–26.8)
Pancreas	2.66	(0.73–6.82)	0.89	(0.02–4.95)	0.00	(0.00–1.54)
Non-Hodgkin lymphoma	1.49	(0.18–5.39)	0.00	(0.00–3.79)	0.52	(0.01–2.87)
Bile ducts, other biliary	1.89	(0.39–5.52)	0.00	(0.00–3.11)	3.12^#^	(1.35–6.14)
Stomach	0.29	(0.04–1.05)	0.99	(0.32–2.31)	0.48	(0.16–1.12)
Liver	1.02	(0.12–3.69)	0.66	(0.02–3.69)	0.63	(0.08–2.29)
Gallbladder	1.24	(0.03–6.93)	1.68	(0.04–9.39)	0.00	(0.00–2.90)
Small intestine	0.00	(0.00–18.16)	6.62	(0.17–36.87)	0.00	(0.00–11.87)
All excluding POFT	1.72^#^	(1.44–2.05)	1.16	(0.89–1.50)	1.28^#^	(1.07–1.52)

*Stage data was used since 2006

POFT: peritoneal, epithelial ovarian, and fallopian tubal; SIR: standardised incidence ratio; CI: confidence interval

^#^significant at α = 0.05

**Fig. 1:**
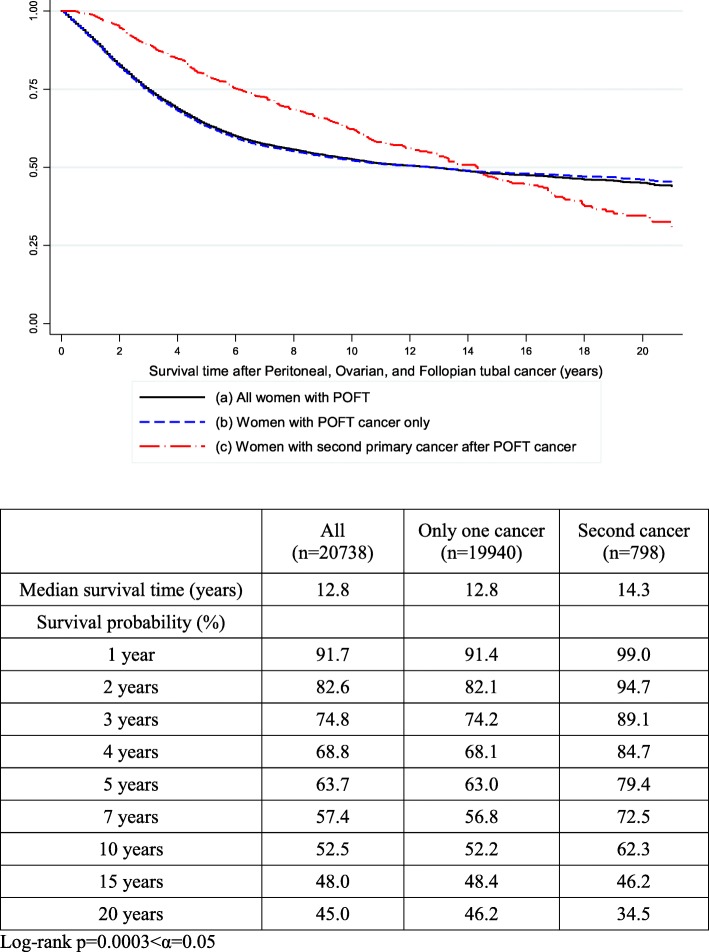
Survival outcomes from the onset of peritoneal, ovarian, and fallopian tubal cancer. All women with peritoneal, ovarian, and fallopian tubal cancer (**a**); women with only peritoneal, ovarian, and fallopian tubal cancer (**b**); women with peritoneal, ovarian, and fallopian tubal cancer and with any second primary cancer (**c**)

**Fig. 2:**
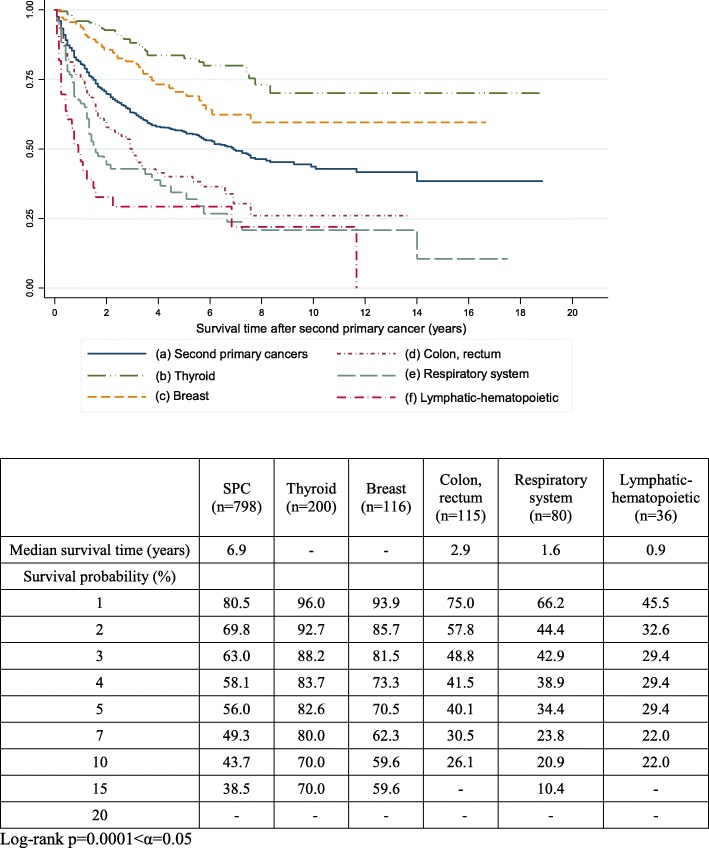
Survival outcomes in peritoneal, ovarian, and fallopian tubal cancer patients with a second primary cancer. Survival time from the onset of any second primary cancer (**a**), thyroid (**b**), breast (**c**), colon (**d**), respiratory system (**e**), and lymphatic-hematopoietic (**f**) second primary cancers

The median overall survival time was 12.8 years in all women with POFT cancer. In POFT cancer women with an SPC, the median overall survival time was 14.3 years. The 5-year overall survival rates were 88.8, 74.4, and 44.2% among patients with localized, regional, and distant stage disease, respectively (Additional file 1: Figure S1). The 5-, 10-, and 20-year overall survival rates after the diagnosis of POFT cancer (Fig. 1) were respectively 63.7, 52.5, and 45% in POFT survivors; 63.0, 52.2, and 46.2% in POFT cancer survivors without an SPC; and 79.4, 62.3, and 34.5% in survivors with an SPC. Figure 1 shows the survival curve of women with SPCs compared to women with POFT cancer but without SPCs. Figure 2 shows the survival outcomes according to the site of SPC after the diagnosis of SPC in women with POFT cancer. In women with thyroid or breast cancer, the median overall survival was not reached and the 5-year overall survival rates were good (82.6 and 70.5%, respectively) compared to that in women with SPCs (56.0%) and all women with POFT cancer irrespective of the development of an SPC (63.7%). The median overall survival times were 2.9, 1.6, and 0.9 years in women with colorectal cancer, respiratory cancer, and hematopoietic malignancies, respectively.

## Discussion

In the current study, the overall SIR for an SPC in survivors after POFT was 1.29 (95% CI, 1.21–1.38) compared to the risk of SPCs in the general population. The risk of SPC increased in young women (SIR, 1.47) and after 10 years follow-up after the initial diagnosis of POFT cancers (SIR, 1.33–1.38). Shared aetiology of the POFT cancer and SPC could be identified in the current study, with ascending colon cancer (1.77), transverse colon cancer (2.43), and breast cancer (SIR, 1.26) being common SPCs. Leukaemia as an SPC, likely resulting from sequelae of active treatment for POFT, was also increased (3.07). The 5-year overall survival rates were 88.8, 74.4, and 44.2% among patients with localized, regional, and distant stage disease, respectively. These rates are comparable to those observed among patients in the United States (92.6, 72.9, and 28.0%, respectively) [6].

POFT cancers are well established hereditary cancers based on mutations in *BRCA1*, *BRCA2,* and mismatch repair genes [7–9]. The life-time penetration of ovarian cancer is quite different depending on the type of genes involved. Recently, the cluster regions within *BRCA1* and *BRCA2* associated with specific cancers have been well established [10]. This means that the life-time risk of specific cancers can be discussed with patients based on their age, familial history, tumour location, type of gene, and specific cluster regions. Even though genetic information was not available in this study, the risk of SPCs after POFT cancer based on the hereditary background could be estimated and investigated. The risk of breast cancer increased 1.26 times, and in young women (aged < 60 years), the risk was 1.3. Further, in women with POFT cancer who had serous histology, the risk of breast cancer as an SPC was 1.58 (Table 3). Serous histology is one of the clinical markers suggesting hereditary predisposition for ovarian cancer [11]. The hereditary predisposed sites of colon cancer risk also increased, including ascending colon (2.26) and transverse colon (4.07) cancers, in POFT cancer survivors aged < 60 years. The increased risk of breast cancer remained for 10 years after the diagnosis of POFT cancer (Table 2). Regarding colon cancer, the risk increased within 5 and 10 years after the diagnosis of POFT cancer. One of the reasons for the increased incidence of colon cancer at 5–10 years after the diagnosis of POFT cancer is that as a baseline work-up of POFT cancer, colonoscopy should be done according to the national insurance guidelines. This results in colon cancer and high-risk colonic polyps frequently being diagnosed together with POFT cancer, and such patients are enrolled in a continuous active surveillance program. Therefore, an individualised surveillance program for these two cancers, namely breast and colon cancers, could be suggested for survivors with POFT cancer, especially in the era of next-generation sequencing [12].

From the National Cancer Institute’s Surveillance, Epidemiology and End Results database, the overall incidence of therapy-related acute myeloid leukaemia is 0.17% (109/63,359) [13]. The median survival time from the diagnosis is 3 months. In the current study, the risk of leukaemia as an SPC was higher among patients receiving chemotherapy than among those who did not receive chemotherapy. Age > 65 years and development of secondary leukaemia are poor prognostic factors. In the current study, the risk of leukaemia increased in women aged < 60 years (3.86) and during 10 years after the initial diagnosis of POFT cancer (3.16 until 5 years and 3.68 during 6–10 years after the initial diagnosis). After 10 years, the risk of leukaemia did not increase significantly. Serous histology (4.77) and distant stage (4.94) were additional risk factors for leukaemia, as leukaemia as an SPC relates to heavy treatment. The median overall survival time was 0.9 months, and more than half of the POFT cancer women with leukaemia (54.5%) as an SPC died. In a previous study in a Taiwanese population, different chemotherapies were compared and 5-fluorouracil was suggested as an independent risk factor for SPC in the multivariate analysis [14]. In the current study, information on the specific chemotherapy regimen was not available to be analysed, whereas young age, serous histology, and distant stage, which are clinical factors requiring heavy treatment, were identified as risk factors for the development of leukaemia as an SPC after POFT cancer up to 10 years after the initial diagnosis of POFT cancer.

It is an interesting finding that the survival outcome in POFT cancer women with SPCs was better until nearly 14 years after the diagnosis of POFT cancer compared to in women without an SPC (Fig. 1-1). In particular, in POFT cancer women with thyroid and breast cancers as the SPC, the survival outcomes were particularly good compared to those with colorectal cancer, respiratory cancer, and hematopoietic malignancies. The higher number of patients in the subgroups showing good survival outcomes may be the reason for the improved survival outcomes in POFT cancer women with SPCs. Moreover, POFT cancer women with breast cancer as the SPC might have a higher possibility of *BRCA1* or *BRCA2* germline mutation compared to women without breast cancer. In women with a pathogenic germline mutation in *BRCA1* or *BRCA2*, the risks of death after adjustment for clinical variables including age, stage, grade, and histology, were decreased, with hazard ratios of 0.73 for *BRCA1* and 0.49 for *BRCA2* [15]. Although the incidence of thyroid cancer in Korean women is the 2nd highest of all cancers, the risk of mortality is not high compared to that in the general population, with an age-standardised mortality rate of 0.3/100,000 [3]. Subgroups of cancers with good biomarkers and effective targeted therapy or immunotherapy based on hereditary factors could reveal improvements of the survival outcomes in the near future [16].

One of the merits in this study is that all cancers were covered by the national cancer centre registry, and all POFT cancers and SPCs were systemically registered by well-trained medical record administrators who were regularly educated. This means minimisation of selection bias and good reproducibility of this study. However, the applicability of these data to non-Korean populations may be limited. To our knowledge, this is the first study to evaluate the development and types of SPC after POFT cancer, not only ovarian cancer. POFT cancers are more homogenous and reproducible in terms of genetic background and clinical scenarios. On the other hand, one of the limitations in this study is that we used limited clinical variables. Thus, the impact of several clinical variables on the development of SPC could not be evaluated, which could be potentially important in daily clinical practice.

## Conclusions

In conclusion, the risk of SPC is increased in POFT survivors up to 10 years after the diagnosis of POFT and in young survivors (< 60 years). Proximal colon and breast cancers, especially in young POFT cancer survivors, are SPCs suggesting hereditary predisposition. Leukaemia tended to develop in women who were heavily treated, especially in cases of young age, serous histology, and distant stage, suggesting that this SPC is chemotherapy-related. Nevertheless, the survival outcome in POFT cancer women with an SPC was favourable. In particular, the development of thyroid and breast cancers as SPCs in women with POFT cancer suggests favourable prognoses. The results of the present study can be used for the surveillance of POFT cancer survivors and to estimate the prognoses of women with POFT cancer and SPCs.

## Additional file

Additional file 1: **Figure S1.** Survival outcomes from onset of POFT cancer according to stage. (TIF 67 kb)

## Abbreviations

CI: Confidence interval
POFT: Peritoneal, epithelial ovarian, and fallopian tubal
PYR: Person-year at risk
SIR: Standardised incidence ratio
SPC: Second primary cancer
