# Supplementary figures and images for: Second primary cancer after primary peritoneal, epithelial ovarian, and fallopian tubal cancer: a retrospective study
Source: BMC Cancer. 2018 Aug 8;18:800. doi: 10.1186/s12885-018-4700-3 (PMC6083613; doi:10.1186/s12885-018-4700-3)

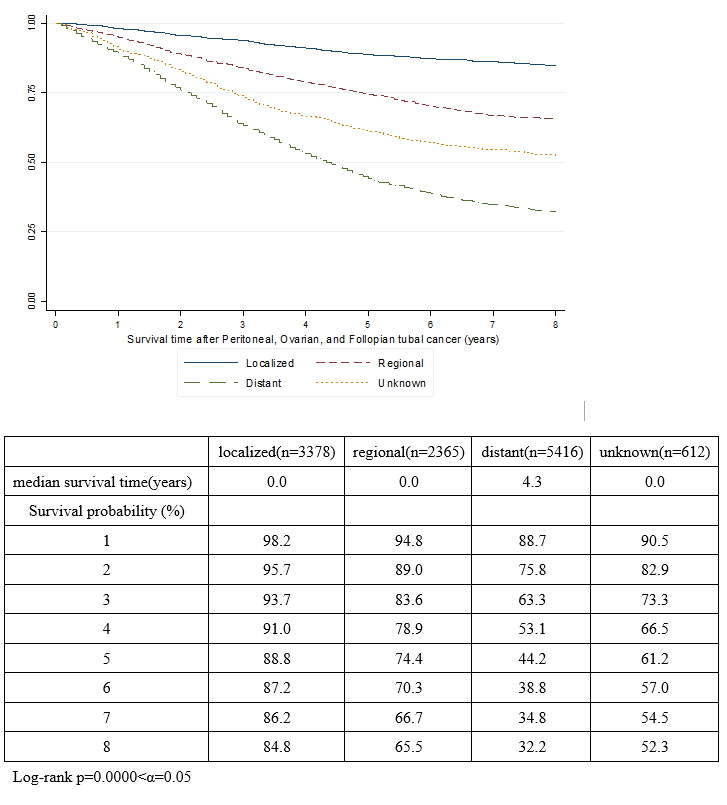

Supplement: Supplementary file 1 — Figure S1. Survival outcomes from onset of POFT cancer according to stage. (TIF 67 kb) [file 12885_2018_4700_MOESM1_ESM.tif]
